# PoCUS-first versus CT-only for non-traumatic abdominal pain: a propensity score-weighted cohort study on ED resource utilization

**DOI:** 10.1007/s11739-025-04164-2

**Published:** 2025-10-27

**Authors:** Te-Fa Chiu, Tse-Chyuan Wong, Fen-Wei Huang, Eric H. Chou, Jon Wolfshohl, Kuan-Fu Chen, Wei-Jun Lin, Shih-Hao Wu

**Affiliations:** 1 Department of Emergency Medicine, China Medical University Hospital, No. 2, Yude Rd, Taichung, 404 Taiwan; 2 School of Medicine, College of Medicine, China Medical University, Taichung, Taiwan; 3 Departments of Emergency Medicine, Baylor Scott and White All Saints Medical Center, Fort Worth, TX USA; 4 Department of Artificial Intelligence, College of Intelligent Computing, Chang Gung University, Taoyuan, Taiwan; 5 Department of Emergency Medicine, Chang Gung Memorial Hospital, Keelung, Taiwan; 6 College of Public Health, China Medical University, Taichung, Taiwan

**Keywords:** Ultrasound, Clinical approach, Mortality, Intensive care unit, Computed tomography, ED crowding

## Abstract

Point-of-care ultrasound (PoCUS) may reduce emergency department (ED) length of stay (LOS) for selected abdominal diagnoses, but its role in undifferentiated, non-traumatic abdominal pain remains uncertain. This study compared PoCUS-first and CT-only strategies, evaluating associations with resource use and clinical outcomes in adult ED patients. This propensity score-weighted cohort study included adults (≥ 18 years) presenting with non-traumatic abdominal pain to the ED of a tertiary medical center in Taiwan between January 2021 and December 2023. Patients transferred or discharged against medical advice from other facilities were excluded. Encounters were classified as PoCUS-first (PoCUS alone and followed by CT) or CT-only. Primary outcomes were ED LOS and costs; secondary outcomes were unscheduled return visit (URV), hospital LOS, total costs, ICU admission, and in-hospital mortality. Multivariate regression and inverse probability of treatment weighting (IPTW) adjusted for baseline differences. Among 26,403 index ED visits, 67.5% received PoCUS‑first evaluation and 32.5% underwent CT only. After adjustment, PoCUS‑first was associated with substantially shorter ED LOS (ratio of means 0.53, 95% CI 0.52–0.54; 47% reduction), lower ED costs (0.52, 95% CI 0.51–0.53; 48% reduction), fewer consultations, and reduced admission rates. In patients discharged at the index visit (n = 17,390), PoCUS‑first was similarly linked to shorter LOS (0.61, 95% CI 0.60–0.62; 39% reduction), markedly lower costs (0.47, 95% CI 0.46–0.47; 53% reduction), and decreased odds of unscheduled return visits (OR 0.82, 95% CI 0.69–0.96), without an increase in adverse outcomes. For patients later admitted after a return visit, PoCUS‑first encounters were characterized by lower resource use during the index ED visit, with no evidence of prolonged subsequent care or safety concerns. A PoCUS‑first strategy, used for initial risk stratification, was associated with greater ED efficiency and more judicious resource use without compromising safety. However, the observed reduction in ED LOS may partly reflect local workflow and in‑house processes, and residual confounding cannot be excluded. Confirmation in prospective, multicenter studies is warranted.

## Introduction

Non-traumatic abdominal pain accounts for approximately 7–10% of all emergency department (ED) visits [1, 2] and contributes to approximately 23% of unscheduled 72-h returns [3]. Establishing a definitive diagnosis in these cases in the ED is challenging, with 32–53% remaining uncertain [4, 5]. The causes of abdominal pain range from mild, self-limiting conditions to severe, life-threatening surgical emergencies [6].

The selective use of bedside ultrasonography (or insonation), combined with traditional inspection, palpation, percussion, and auscultation, has become one of the five pillars of bedside clinical medicine [7]. Point-of-care ultrasonography (PoCUS) is a bedside diagnostic tool that enables clinicians to rapidly acquire, interpret, and integrate ultrasound findings into patient care [8], as endorsed by major professional guidelines, including those from the American College of Emergency Physician [9] and the Society of Critical Care Medicine [10]. It demonstrates high sensitivity and specificity for certain conditions [11] and can enhance diagnostic accuracy while guiding management decisions [12, 13]. Several studies also show PoCUS can shorten ED length of stay (LOS), particularly in renal colic [14, 15], cholecystitis [16], diverticulitis [17], and small bowel obstruction [18]. However, evidence for its broader application in undifferentiated, non-traumatic abdominal pain remains limited.

The benefits of PoCUS should be weighed against other diagnostic modalities. Routine computed tomography (CT) provides superior diagnostic confidence for surgical decision-making in acute abdominal pain [19] and has been shown to improve diagnostic accuracy in a meta-analysis [20]. Despite these strengths, CT is associated with increased cost and radiation exposure, making PoCUS a potentially valuable alternative [18]. The accuracy of PoCUS, however, is highly operator-dependent [21], influenced by patient characteristics, clinician expertise, and equipment quality [22]. To address this variability, international guidelines emphasize the need for structured education, supervised hands-on training, and formal competency assessments [9]. Sustained proficiency also requires ongoing practice and refresher training, as skills may decline without regular use.

Return visits to the ED, typically defined as occurring within 72 h [23], are a key quality indicator of patient safety [24] and clinical care [25]. Return visit rates range from 0.4 to 4.9% [23], with up to one‑third considered avoidable [26]. Such revisits worsen ED overcrowding [27], which in turn is linked to higher mortality, diminished care quality, and increased healthcare costs [28]. Although most patients report satisfaction with the discharge process, many voice concerns about inadequate assessment or treatment during the initial visit [29]. In this context, the perceived quality and safety of PoCUS may strongly influence emergency physicians’ willingness to adopt it in routine practice.

Most prior PoCUS research has examined diagnostic accuracy [30, 31], LOS, or ED costs for specific diagnoses [11, 32], without assessing its broader impact in patients presenting with non‑traumatic abdominal pain. One trial found that systematic PoCUS did not improve diagnostic accuracy in unselected ED patients [33]. The effect of widespread PoCUS adoption on the safety of this patient group therefore remains uncertain. We hypothesized that a PoCUS‑first strategy would shorten ED LOS, lower costs, reduce unplanned readmissions, and maintain patient safety and quality of care. This study compared PoCUS-first and CT-only strategies in adult ED patients with non‑traumatic abdominal pain, focusing on differences in resource use as well as patient outcomes.

## Methods

### Study design

This propensity score–weighted cohort study was conducted at a tertiary medical center in Taiwan with > 160,000 annual ED visits. Electronic medical records (EMR) from 1 January 2021 to 31 December 2023 were analyzed. The institutional review board approved the study protocol (CMUH113‑REC2‑008), and informed consent was waived. STROBE guidelines for observational studies were followed, and all elements in the checklist for cross-sectional studies are presented in content and structure [34].

### Setting and population

This study builds on previous research to examine the performance of the PoCUS-first strategy [35]. The patients were included if they (i) had an ED visit with no prior ED visit or hospitalization in the preceding three days; (ii) had non-traumatic abdominal pain as the chief complaint at triage; (iii) were adults aged 18 years or older. Patients were excluded if they: (i) were younger than 18 years; (ii) presented with traumatic abdominal pain; (iii) had been transferred from, or discharged against medical advice by, another facility; or (iv) did not receive PoCUS or CT during the index visit. This study included 26,403 patients.

#### PoCUS practice and training

PoCUS has been embedded in Taiwan’s emergency medicine residency curriculum since 2012. All residents passed a standardized hands-on skills assessment under instructors certified by the Taiwan Society of Ultrasound in Medicine, each with > 10 years of sonography experience. Both residents and attending physicians perform PoCUS, supported by regular refresher courses and monthly meetings to review image quality. These measures help maintain proficiency, minimize inter-operator variability, and optimize the clinical utility of PoCUS.

#### Imaging modality selection

The decision to use PoCUS or CT was left to the treating physician’s clinical judgment, considering experience, patient flow, and the clinical environment (flowchart of the clinical pathway was shown in Supplementary Figure S1).

#### Outcome definitions

A return visit was defined as an ED revisit within 72 h after discharge from the index visit; only the first revisit was analyzed. We focused on early revisits/readmissions, which are more preventable and amenable to hospital-based interventions [36].

#### Study groups

Patients were classified into four groups based on imaging at the index visit: (1) no PoCUS or CT; (2) PoCUS alone; (3) PoCUS followed by CT in the same visit; (4) CT alone. For analysis, groups 2 and 3 were combined as the PoCUS-first cohort and compared with the CT-only cohort to assess the association of initial PoCUS with ED LOS, costs, and quality-of-care indicators, including unscheduled return visits (URV) with admission.

Sixteen diagnostic groups were defined a priori based on a literature review [37] and clinical consensus. Group assignment used the first five ICD‑10 discharge diagnosis codes from the index visit; patients could be assigned to multiple groups if applicable.

### Variables

The EMR of the study center includes patient demographics, visit date and time, triage level, comorbidities, diagnostic codes (ICD-10-CM), imaging (PoCUS, CT), consultations, ED disposition, ED LOS, costs, and hospital LOS.

For this study, a consultation was defined as a formal request by the ED physician for in-person evaluation by a specialty or surgical service (e.g., gastroenterology, trauma and acute care surgery) to assist with diagnosis, intervention, or admission decisions.

Triage level was recorded using the Taiwan Triage and Acuity Scale, a computerized five-level system (Level 1 = resuscitation; Level 5 = non-urgent) [38].

### Outcome measures

Primary outcomes were ED LOS, ED costs. Secondary outcomes were in-hospital mortality, ICU admission, hospital LOS, and total ED + inpatient costs (New Taiwan dollars, NT$). The outcomes were also examined in the subgroup of patients discharged from the index ED visit, which provided valuable additional information on safety.

ED LOS was defined as the interval from triage to ED departure, measured at one of five disposition points: discharge from ED, discharge from observation, admission to general ward, admission to ICU, or ED death. Hospital LOS was recorded for admitted patients to assess prognosis.

### Data analyses

Continuous variables are presented as mean ± standard deviation (SD) or median (interquartile range, IQR), depending on distribution, and compared using Student’s t test or the Mann–Whitney U test. Categorical variables are expressed as counts (percentages) and compared using the Chi-square test. Analyses compared the PoCUS-first group with the CT-only group, and the PoCUS group (with or without CT) with the no-PoCUS group. Missing data were confined to vital signs and anthropometric measures (weight, height, and BMI). Cases with missing values for these variables were excluded from the corresponding analyses and summary statistics; no imputation was performed. The overall proportion of missingness was minimal (< 1% across all key variables, *supplementary table S1*).

To account for confounding, we fitted generalized linear models with a Gamma distribution and log link (appropriate for right-skewed data), adjusting for age, sex, triage level, body mass index (BMI), and comorbidities. To mitigate selection bias, we applied inverse probability of treatment weighting (IPTW) using propensity scores derived from the same covariates (age, sex, triage level, BMI, and comorbidities), using stabilized weights to limit extreme values. Covariate balance was evaluated using standardized mean differences, with values < 0.1 indicating negligible imbalance and 0.1–0.2 suggesting minimal imbalance.

Group differences in LOS and costs were analyzed in the IPTW-adjusted cohort using Gamma regression. Results were expressed as ratios of means (RMs) or odds ratios (ORs) with 95% confidence intervals (CIs), obtained by exponentiating the model coefficients (*exp*β). An RM greater than 1 indicates a proportional increase in the expected outcome, whereas an RM less than 1 indicates a decrease. All statistical analyses were performed using SAS software (version 9.4; SAS Institute Inc., Cary, NC, USA). Two-sided P values < 0.05 were considered statistically significant.

## Results

### Study population

#### Distribution

**Fig. 1:**
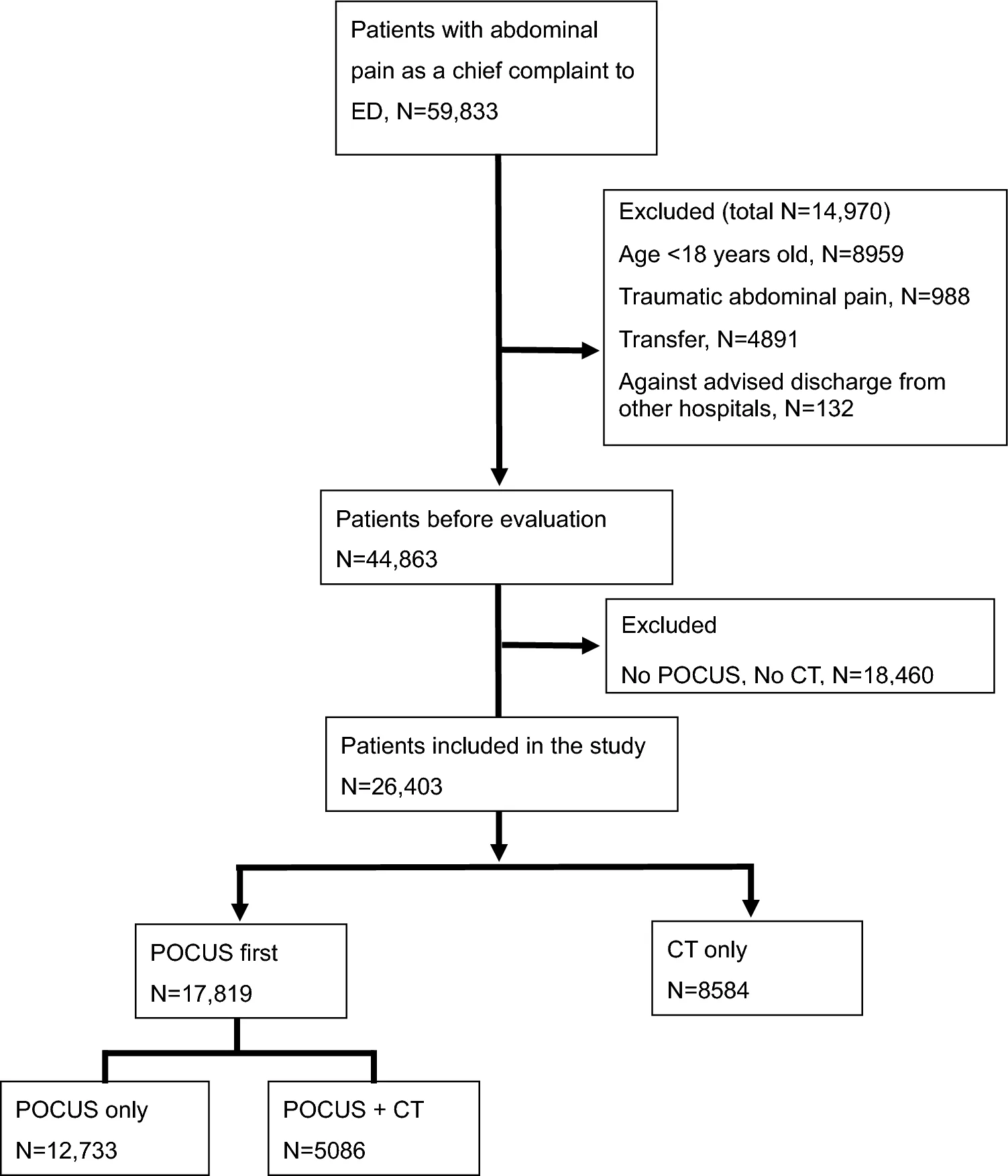
Flow diagram of study population selection

26,403 index ED visits were entered in the core analyses (Fig. 1). Of them, 17,819 patients (67.5%) underwent PoCUS‑first and 8584 (32.5%) underwent CT‑only.

#### Baseline demographics

Table 1 summarizes baseline demographics and clinical characteristics at the index visit. Compared with the CT-only group, patients in the PoCUS-first group were significantly younger, more often female, and more frequently triaged as less urgent. After adjustment, they still had shorter ED LOS, lower ED costs, and fewer consultations, hospital admissions, ICU admissions, and in-ED deaths. Conversely, the PoCUS-first group had a higher proportion of outpatient disposition and more URVs. These patterns suggest that patients in the CT-only group generally presented with greater disease severity than those in the PoCUS-first group.

**Table 1:** Demographics of the study population at the index visit

	Data before IPTW			Data after IPTW		
	PoCUS-first N = 17,819 (67.5%)	CT N = 8584 (32.5%)	p	PoCUS-first	CT	SMD
Age	42.91 ± 17.80	53.16 ± 19.29	< 0.001	46.36 ± 23.31	46.4 ± 32.99	-0.001
*Sex*			< 0.001			
M	6497(36.46)	4068(47.39)		6497(40.34)	4068(41.03)	-0.024
F	11,322(63.54)	4516(52.61)		11,322(59.66)	4516(58.97)	0.016
BMI	24.11 ± 24.98	24.68 ± 43.60	0.257	24.34 ± 37.69	24.4 ± 66.76	-0.001
*Triage*			< 0.001			
1	104(0.58)	211(2.46)		104(1.21)	211(1.18)	0.080
2	1684(9.45)	1640(19.11)		1684(12.67)	1640(12.56)	0.012
3	15,750(88.39)	6638(77.33)		15,750(84.70)	6638(84.86)	-0.002
4	279(1.57)	94(1.10)		279(1.41)	94(1.39)	0.043
5	2(0.01)	1(0.01)		2(0.01)	1(0.01)	-0.005
HR	87.43 ± 17.29	91.76 ± 19.23		87.06 ± 21.43	92.35 ± 32.75	-0.191
SBP	130.2 ± 23.93	132.3 ± 25.72		131.9 ± 30.38	129.7 ± 41.97	0.061
DBP	80.67 ± 14.61	79.64 ± 13.94		80.74 ± 18.33	79.81 ± 23.36	0.044
BT	36.63 ± 0.68	36.79 ± 0.89		36.62 ± 0.85	36.83 ± 1.50	-0.172
RR	19.47 ± 1.56	19.64 ± 1.60		19.49 ± 1.92	19.59 ± 2.69	-0.042
Comorbidities	8392(47.10)	5349(62.31)	< 0.001	52.02%	51.45%	0.016
*ED resource utilization*						
LOS in ED (hr)	2.90(1.80–5.40)	6.60(4.20–13.10)	< 0.001	3.00(1.90–5.80)	6.00(3.90–11.20)	-0.297
ED cost	3925(2816–9021)	10,982(9355–13,382)	< 0.001	4125(2903–9537)	10,494(9140–12669)	-0.489
Consultation	3995(22.42)	3896(45.39)	< 0.001	3995(23.03)	3896(43.01)	-0.869
Discharge with OPD	13,791(77.39)	3599(41.93)	< 0.001	13,791(75.10)	3599(47.33)	0.646
Admission	2406(13.50)	3778(44.01)	< 0.001	2406(15.28)	3778(39.61)	-1.277
ICU	69(0.39)	135(1.57)	< 0.001	69(0.60)	135(1.05)	-1.692
Expire in ED	1(0.01)	13(0.15)	< 0.001	1(0.01)	13(0.08)	-0.488
Unscheduled return visit	632(3.55)	223(2.60)	< 0.001	632(3.68)	223(2.66)	-0.546

*IPTW* inverse probability of treatment weighting, *SMD* standardized mean difference, *PoCUS* point-of-care ultrasonography, *CT* CT only, *LOS* length of stay, *ED* emergency department, *ICU* intensive care unit, *OPD* outpatient disposition

### Subgroup analysis of the primary outcome by diagnosis in the whole cohort

**Fig. 2:**
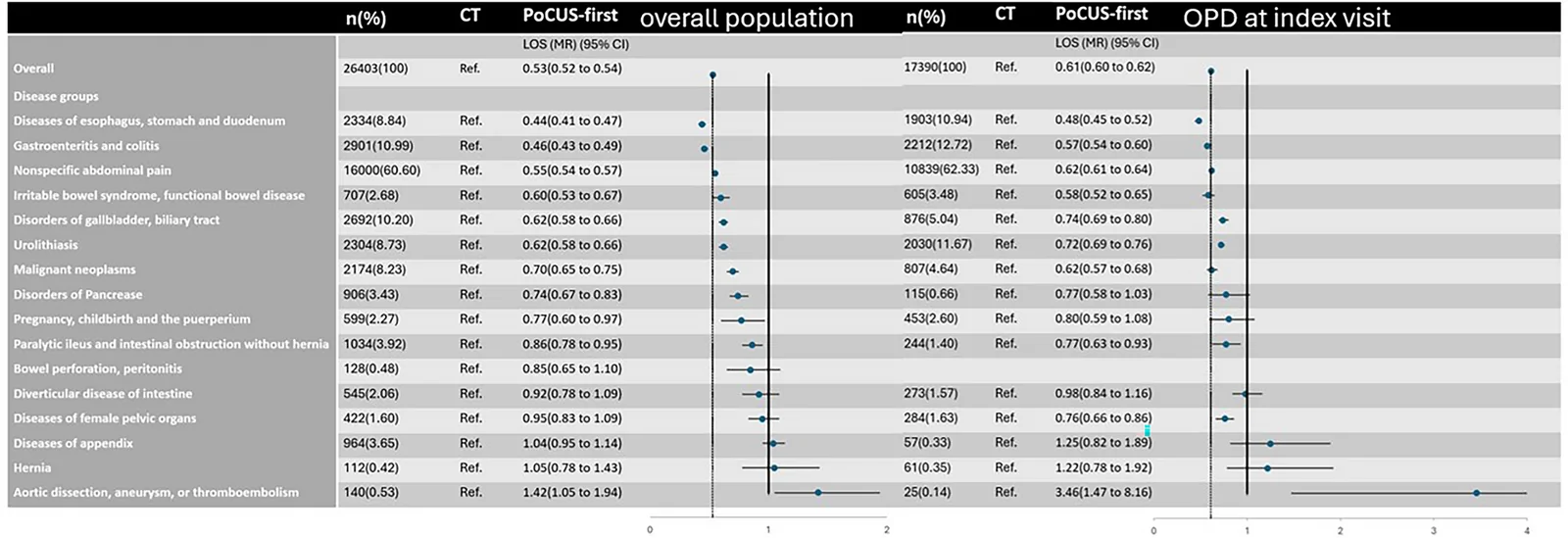
The Subgroup analysis of ED LOS with PoCUS‑first versus CT‑first

As shown in Fig. 2, in the IPTW analysis, PoCUS‑first evaluation was associated with consistently shorter ED LOS compared with CT‑first across the majority of diagnostic subgroups.

For the overall population (n = 26,403), PoCUS-first was associated with a 47% reduction in LOS relative to CT-first (ratio of means 0.53, 95% CI 0.52–0.54), and lower ED costs (0.52, 95% CI 0.51–0.53; 48% reduction). The effect was most pronounced in high‑volume conditions such as non-specific abdominal pain, gastroenteritis/colitis, and upper gastrointestinal disorders, where LOS was reduced by 40–55%. Moderate reductions were also observed in biliary tract disease, urolithiasis, and malignant neoplasms.

Infrequent diagnoses demonstrated heterogeneous effects: pancreatic and pregnancy‑related presentations showed variable results, while appendicitis, hernia, and diverticular disease showed no significant differences. Notably, PoCUS‑first was associated with longer LOS in patients with aortic dissection, aneurysm, or thromboembolism. These findings suggest that PoCUS‑first strategies may improve ED efficiency for common abdominal presentations; however, in conditions where CT confirmation is essential for diagnosis, or where patients—although not requiring immediate surgery—must remain in the ED for observation or await transfer to a ward or ICU, the impact of PoCUS on LOS appears limited or less favorable.

Forest plot shows relative ED LOS (95% CI) in the IPTW cohort, stratified by diagnostic category for the overall population and outpatients discharged at the index visit. PoCUS‑first was associated with shorter LOS in most common abdominal presentations (e.g., non-specific abdominal pain, gastroenteritis/colitis, upper GI disorders), whereas no significant differences were observed in appendicitis, hernia, or diverticular disease, and longer LOS was noted in aortic emergencies.

### Study outcomes in patients discharged with OPD at the index visit

#### Primary outcomes

As shown in Table 2, among patients discharged with OPD at the index visit, PoCUS‑first was consistently associated with reduced ED resource utilization compared with CT‑only, both before and after IPTW adjustment. The mean ED LOS for PoCUS‑first was 0.60 (95% CI, 0.59–0.62) before IPTW and 0.61 (95% CI, 0.60–0.62) after IPTW, representing approximately 40% shorter stays than CT (both p < 0.001). ED costs were also markedly lower with PoCUS‑first, at 0.48 (95% CI, 0.47–0.49) before IPTW and 0.47 (95% CI, 0.46–0.47) after IPTW, corresponding to about a 50% reduction (both p < 0.001). PoCUS‑first also showed lower consultation rates and reduced odds of an URV. These indicated that initial PoCUS use may expedite care and reduce repeat ED visits.

**Table 2:** Adjusted outcomes in patients discharged with OPD at The Index Visit

Outcome measures, RM or OR (95% CI)*	CT	Data before IPTW		Data after IPTW	
		PoCUS-first	p value	PoCUS-first	p value
LOS in ED (hr)	Ref	0.60 (0.59–0.62)	< 0.001	0.61 (0.60–0.62)	< 0.001
ED cost	Ref	0.48 (0.47–0.49)	< 0.001	0.47 (0.46–0.47)	< 0.001
Consultations	Ref	0.48 (0.44–0.53)	< 0.001	0.53 (0.48v0.58)	< 0.001
Unscheduled return visit	Ref	0.83 (0.71–0.98)	0.026	0.82 (0.69–0.96)	0.014
OPD after an URV	Ref	0.88 (0.69–1.11)	0.279	0.85 (0.67–1.09)	0.200
Admission after an URV	Ref	0.95 (0.74–1.22)	0.708	0.92 (0.72–1.19)	0.538
ICU admission after an URV	Ref	1.09 (0.19–6.28)	0.925	0.91 (0.15–5.65)	0.921
Expire in ED after an URV	Ref	< 0.01 (< 0.01– > 999.9)	0.730	0.001 (< 0.01–0.01)	< 0.001

Data are presented as Gamma distribution with log link (handles right-skewed data). *RM* ratio of means, *OR* odds ratio, *IPTW* inverse probability of treatment weighting, *PoCUS* point-of-care ultrasonography, *CT* CT only, *URV* unscheduled return visit, *OPD* outpatient disposition

#### Secondary outcomes

Rates of outpatient management after an URV, admission after an URV, and ICU admission after an URV did not differ significantly between groups. Mortality at return visits was rare, with no signal of harm in the PoCUS-first group. Together, these findings indicate that efficiency gains from a PoCUS-first approach were achieved without compromising safety or appropriateness of care.

### Study outcomes in patients admitted after an URV

#### Primary outcomes

As shown in Table 3, among patients admitted following an URV, the PoCUS‑first group was associated with a significantly shorter index ED LOS and lower index ED costs compared with the CT‑only group, both before and after IPTW adjustment. No statistically significant differences were observed in total ED LOS or total ED cost.

**Table 3:** Adjusted outcomes using IPTW in patients admitted after unscheduled ED return visit

Outcome measures, RM (95% CI)	CT	Data before IPTW		Data after IPTW	
		PoCUS-first	p	PoCUS-first	p
LOS in 1^st^ED	Ref	0.76 (0.66–0.87)	< 0.001	0.76 (0.67–0.85)	< 0.001
Total ED LOS	Ref	0.92 (0.79–1.07)	0.256	0.90 (0.79–1.03)	0.123
1^st^ED cost	Ref	0.52 (0.44–0.60)	< 0.001	0.51 (0.45–0.57)	< 0.001
2nd ED cost	Ref	1.57 (1.32–1.88)	< 0.001	1.59 (1.37–1.84)	< 0.001
Total ED cost	Ref	1.11 (0.94–1.30)	0.215	1.12 (0.98–1.28)	0.108
Admission costs	Ref	1.18(0.96–1.44)	0.119	1.20 (0.99–1.43)	0.052
Total costs	Ref	1.13 (0.96–1.33)	0.153	1.14 (0.99–1.31)	0.070
Hospital LOS	Ref	0.90(0.76–1.06)	0.202	0.89 (0.77–1.03)	0.106
Expire, OR	Ref	–	–	–	–
ICU, OR	Ref	–	–	–	–

Adjusted for age, gender, triage, BMI, and comorbidities. *RM* ratio of means, *IPTW* inverse probability of treatment weighting, *PoCUS* point-of-care ultrasonography, *CI* confidence interval, *NT$* new Taiwan dollar, *CT* computer tomography, *Ref*. reference, *ED* emergency department, *LOS* length of stay, *Total costs* total ED costs + admission costs

#### Secondary outcomes

No significant between-group differences were found in admission costs, total medical cost (ED + admission), hospital LOS, in-hospital mortality, or ICU admission rates during the return admission. These findings demonstrate that, even among patients admitted after an URV, a PoCUS-first strategy can reduce initial ED resource use without prolonging subsequent care or compromising safety outcomes.

## Discussion

In this large, propensity score-weighted cohort of over 26,000 adult ED visits for non‑traumatic abdominal pain, a PoCUS-first approach was consistently associated with shorter ED LOS and over 50% lower costs, without observable safety concerns. These associations were evident across discharged patients, those admitted after unscheduled return visits, and individuals who underwent CT during the index encounter. The findings provide high-quality, real-world insights into the use of PoCUS within a high-volume, resource-intensive emergency care setting.

### Decisions to perform PoCUS

Before interpreting these findings, it is crucial to address the principal limitation of this observational design: confounding by indication. The decision to perform PoCUS first is not random. In this study, PoCUS was applied broadly to address clinical questions arising during patient care, often requiring multi-system assessment based on presenting symptoms, rather than being limited to a single organ or predefined diagnosis. All physicians had standardized PoCUS training and could use any learned protocol or technique to guide decisions. This choice was driven by physician gestalt, which integrated patient-specific factors (clinical acuity, suspected pathology, and body habitus), clinician experience, and system-level constraints like departmental workload. Consequently, a PoCUS-first approach was likely favored for more stable patients with focused diagnostic questions, such as suspected cholelithiasis or hydronephrosis. In contrast, patients who appeared more critically ill or presented with diffuse, alarming symptoms were probably sent directly to CT for a rapid, comprehensive evaluation. While our propensity score model adjusted for numerous measured covariates to mitigate this bias, it cannot fully account for the unmeasured clinical intuition that guides these critical decisions. This unmeasured gestalt remains a significant potential confounder. These include operator experience and skill with PoCUS, subtle clinical cues not captured by triage level or ICD codes, and physician-specific factors such as risk tolerance and decision-making style. For example, more experienced clinicians may achieve greater diagnostic efficiency with PoCUS, while more risk-averse physicians may favor CT, potentially prolonging LOS independent of patient severity. These factors could bias the observed associations in either direction. A directed acyclic graph (DAG) has been included in the Supplementary Materials to clarify the conceptual framework and assumptions underlying our analysis *(Supplementary Figure S2).*

Therefore, our findings should be interpreted as an association with a specific care pathway rather than a direct causal effect of the PoCUS modality itself. However, this limitation is also a strength: the study’s design mirrors the reality of clinical decision-making in emergencies, enhancing the generalizability of our observations.

### PoCUS as a tool for workflow optimization in undifferentiated abdominal pain

Our findings indicate that a PoCUS-first approach was consistently associated with substantial reductions in ED LOS and resource utilization. Notably, this pattern persisted even among patients who ultimately underwent CT imaging; the “PoCUS-then-CT” pathway was significantly faster than a “CT-only” strategy. These observations suggest that CT availability may represent an operational bottleneck, and that early bedside PoCUS may help navigate such delays. By providing immediate diagnostic clues, such as the presence of free fluid, gallstones, hydronephrosis, or bowel dilatation. PoCUS may support more timely refinement of differential diagnoses, initiation of treatment, and selection of definitive imaging [39].

This study extends the conclusions from previous disease-specific research (e.g., in renal colic [14, 15], and cholecystitis [16]) to the much broader, undifferentiated population of patients with non-traumatic abdominal pain, a scenario that better reflects the diagnostic uncertainty of real-world emergency medicine. While prior trials have often been limited by single-operator designs [40], a small group of certified physicians [33], or simulation models [18], our analysis draws from a large-scale, standardized ED environment. These findings offer generalizable, real‑world insights into how PoCUS may be integrated as a risk‑stratification tool to complement, rather than replace, advanced imaging across a wide spectrum of patients.

### The role of PoCUS for non-traumatic abdominal pain

These results contrast with a prior randomized trial in unselected ED patients, which found no improvement in diagnostic accuracy with routine PoCUS [33]. Like all imaging modalities, PoCUS carries risks of false positives and negatives, with accuracy influenced by patient factors, operator skill, and equipment quality [22, 30]. However, in emergency care, PoCUS is not intended as a definitive diagnostic tool, but rather as a rapid screening adjunct to guide clinical decisions and identify critical conditions [7, 8, 41]. In this study, CT was readily available when PoCUS findings were inconclusive or when surgical confirmation was deemed necessary. Accordingly, this study did not assess diagnostic accuracy, but instead examined PoCUS as a screening tool embedded within routine emergency department workflows, addressing a critical gap in evidence regarding its practical integration.

### Patient safety or quality of care in the PoCUS-first group

In this cohort, a PoCUS‑first approach was associated with higher rates of OPD and lower hospital admission rates, without observable safety concerns. ICU admission, mortality, and adverse event rates were comparable between groups, and ED mortality was numerically lower in the PoCUS group. These safety signals remained consistent even after accounting for unscheduled return visits. Although CT use for abdominal pain continues to rise [42], only 2.5% of patients who received both PoCUS and CT were admitted after an URV, identical to the CT‑only group, suggesting that such revisits may more often reflect unpredictable disease progression than deficiencies in the initial care. Prior studies have shown that return visits for non‑specific abdominal pain can lead to clinically meaningful changes in diagnosis or management [43], reinforcing that they are not, in isolation, indicators of suboptimal care [44]. Taken together with the existing literature, these findings support PoCUS as a safe and pragmatic tool for early risk stratification in the ED, particularly in undifferentiated, non‑traumatic abdominal presentations.

### Generalizability and workflow differences

The generalizability of our findings may be influenced by differences in PoCUS availability, training, and workflow across health systems. In some institutions, ultrasound machines may be outdated, poorly maintained, or not readily accessible due to space constraints or crowded environments, limiting the feasibility of routine PoCUS use [45]. In addition, the absence of dedicated scanning areas, cumbersome reporting processes, or insufficient numbers of trained instructors and quality assurance programs may further reduce the consistency and reliability of PoCUS performance [46]. These variations in infrastructure, workflow integration, and training support could lead to differences in diagnostic accuracy and clinical impact compared with our setting, where PoCUS training and implementation are standardized. Therefore, our results should be interpreted with caution when applied to institutions with less supportive environments for PoCUS.

### Strengths and limitations

This study examined a broad spectrum of non-traumatic abdominal pain presentations using a large sample, extended timeframe, and robust statistical adjustments. Several limitations should be noted. *First, as an observational analysis, residual confounding remains a concern despite the use of IPTW based on measured covariates. Important unmeasured factors may have influenced both diagnostic pathway selection and outcomes.* Missing data were minimal (< 1%) and unlikely to have materially altered the observed associations. Second, clinical diagnoses and PoCUS indications were not standardized, reflecting real-world variability and illustrating PoCUS use as a pragmatic risk-stratification tool.* Third, ED LOS may have been shaped by unmeasured operational factors, such as CT waiting times, reporting or consultation delays, and ward availability; however, both groups were exposed to the same institutional environment.* Fourth, findings are limited to adults and may not extend to pediatric populations. Fifth, PoCUS accuracy was not assessed, as imaging decisions relied on clinician judgment consistent with routine practice. Sixth, return visits to other facilities and post-discharge deaths could not be captured, though prior data suggest minimal impact [47]. *Finally, the study reflects the experience of a single center where PoCUS training is standardized and well integrated into workflows. Generalizability may therefore be limited in settings with different levels of operator training or less established PoCUS implementation, where diagnostic performance and clinical pathways could vary.* These limitations underscore the need for prospective, multicenter studies while highlighting the relevance of this analysis for understanding how PoCUS is integrated into emergency care.

## Conclusions

In this observational study, a PoCUS‑first diagnostic approach in the ED was associated with greater front‑end efficiency, lower resource use, and no signal of harm across multiple patient subgroups. These findings indicate that PoCUS may function as a practical initial imaging modality, risk‑stratification tool, and adjunct to physical examination for selected non‑traumatic abdominal presentations. Selective use may help direct CT toward patients most likely to require it, reduce avoidable admissions, and alleviate ED crowding. Given the potential for residual confounding, these associations should be interpreted with caution. Prospective, multicenter randomized trials are needed to confirm these observations and to assess not only efficiency but also diagnostic accuracy for critical conditions and patient‑reported outcomes.

## Data Availability

All relevant materials are presented in the present manuscript. The raw data supporting the conclusions of this article will be made available by the author, Shih-Hao Wu, without undue reservation. E-mail address: ambertwu@gmail.com.
